# *Artemisia annua* and Its Derivatives Improve the Refrigerated Shelf Life of Nile Tilapia Fillets

**DOI:** 10.3390/foods15132387

**Published:** 2026-07-04

**Authors:** Mayumi Fernanda Aracati, Leticia Franchin Rodrigues, Susana Luporini de Oliveira, Romário Alves Rodrigues, Camila Carlino-Costa, Mary Ann Foglio, Marita Vedovelli Cardoso, Hirasilva Borba, Gabriel Augusto Marques Rossi, Jorge Galindo-Villegas, Luiz Arthur Malta Pereira, Marco Antonio de Andrade Belo

**Affiliations:** 1 Department of Pathology, Reproduction and One Health, Sao Paulo State University (UNESP), Jaboticabal 14884-900, SP, Brazil; mayumiaracati@hotmail.com (M.F.A.); leticia.franchin@unesp.br (L.F.R.); susana.luporini@unesp.br (S.L.d.O.); romario.a.rodrigues@unesp.br (R.A.R.); camila.carlino@unesp.br (C.C.-C.); marita.vedovelli@unesp.br (M.V.C.); hirasilva.borba@unesp.br (H.B.); 2 Faculty of Pharmaceutical Sciences, Universidade Estadual de Campinas, Campinas 13083-859, SP, Brazil; maryann.foglio@fcf.unicamp.br; 3 Microbiology Department, Vila Velha University (UVV), Vila Velha 29102-920, ES, Brazil; gabrielrossiveterinario@hotmail.com; 4 Department of Genomics, Faculty of Biosciences and Aquaculture, Nord University, 8020 Bodø, Norway; jorge.galindo-villegas@nord.no; 5 Laboratory of Animal Pharmacology and Toxicology, Brazil University, Descalvado 13690-000, SP, Brazil; luiz.pereira@ub.edu.br

**Keywords:** artemether, artemisinin, oxidative stability, flavonoids, *Oreochromis niloticus*, food safety

## Abstract

*Artemisia annua* contains artemisinin, a sesquiterpene lactone endoperoxide; artemether is a semisynthetic derivative of artemisinin that may offer potential advantages due to its redox-modulating and antimicrobial activities. These compounds have been associated with oxidative-stress modulation and microbial inhibition, making them promising candidates for experimental evaluation in nutritional and post-harvest quality studies. This study evaluated the effect of dietary supplementation with *A. annua* powder, artemisinin, and artemether on the refrigerated quality of Nile tilapia (*Oreochromis niloticus*) fillets. A total of 160 Nile tilapia were randomly assigned to four treatments: control (no additive), 1% *A. annua* powder, artemisinin (9.6 mg/kg feed), or artemether (9.6 mg/kg feed). After 30 days of feeding, 320 fillets were collected and stored under refrigeration at 4 °C. Samples were analyzed immediately after slaughter (day 0) and on days 7, 15, and 30. For each treatment group and sampling time, 20 fillets were used: 10 for microbiological evaluations, including counts of mesophilic and psychrotrophic bacteria, molds and yeasts, sulfite-reducing *Clostridium*, Enterobacteriaceae, coagulase-positive staphylococci, and coliforms; and 10 for physicochemical analyses, including pH, colorimetry, lipid oxidation through TBARS, and sensory evaluation. All supplemented treatments demonstrated improved microbial stability and lower TBARS values when compared with the control. Spoilage indicators such as discoloration, texture loss, and odor deterioration were also delayed. Artemether showed the most pronounced benefits, with lower microbial loads and oxidation indices for several evaluated parameters. These findings suggest that dietary supplementation with *A. annua* and its derivatives may help delay post-harvest quality deterioration of tilapia fillets during refrigerated storage.

## 1. Introduction

Freshness remains one of the most important quality attributes of fish, particularly in refrigerated non-frozen products [1] and it is closely linked to perceived safety, reliability, and superior sensory quality for consumers [2]. Fish are vulnerable to microbiological and chemical deterioration due its intrinsic factors such as high-water activity, neutral pH and high content of polyunsaturated fatty acids (PUFAs) [3]. Under ice storage, fish shelf life can extend from only a few days to nearly three weeks depending on species and handling practices, highlighting the importance of an uninterrupted cold chain to curb microbial proliferation and slow quality deterioration [4]. Thus, strategies aimed at preserving refrigerated fish should target both lipid oxidation and spoilage-associated microbial growth [3,4].

Fish is valued for its high protein content and low saturated fat [2], and the World Health Organization recommends consuming fish one to two times per week to support adequate omega-3 intake [5]. Aquaculture is therefore increasingly central to global food security, generating US$ 296 billion in 2022 [6], reinforcing the broader “Blue Revolution” with food safety and sustainability [7]. Within this context, tilapia stands out as a key species, and Brazil illustrates this trend, *Oreochromis niloticus* is the country’s leading farmed fish due to favorable production traits and an increasingly specialized value chain, placing Brazil as the fourth largest producer worldwide, with more than 707,400 tons in 2025 [8].

Growing consumer concern regarding synthetic preservatives has driven the food industry to seek natural alternatives in plants, animals, bacteria, algae, and fungi [2]. Several studies have focused on natural antioxidants and antimicrobial compounds to extend the shelf life and preserve the quality of fish, protecting lipids against peroxidation and inhibiting oxidant chain reactions [9,10,11]. Widely used in traditional herbal medicine, *A. annua* gained global scientific prominence after Tu Youyou received the Nobel Prize in Medicine 2015. Among its compounds, artemisinin is notable for its sesquiterpene lactone structure containing a rare 1,2,4-trioxane endoperoxide ring [9,12]. However, the biological activity of *A. annua* is not limited to artemisinin, since the plant also contains flavonoids, phenolic acids, terpenoids, and volatile compounds that may contribute to antioxidant and antimicrobial effects [13,14]. In addition to protecting against oxidative deterioration [15] and reducing oxidative stress [16], *A. annua* has antibacterial, antiparasitic, antifungal, antileishmanial, antitumor and anti-inflammatory properties [17,18,19,20]. In Nile tilapia, dietary supplementation with alcoholic extract of *A. annua* has been associated with modulation of gastrointestinal microbiota, improved performance, resistance, immune responses, and antioxidant biomarkers, supporting its evaluation as a candidate dietary supplement [21,22].

The mechanisms by which artemisinin and its derivatives may influence fillet preservation are complex. Phenolic compounds and flavonoids from *A. annua* may contribute to antioxidant protection by reducing reactive oxygen species and limiting lipid peroxidation. In contrast, artemisinin and artemether should not be described only as classical antioxidants, because their endoperoxide bridge can be activated in the presence of Fe^2+^ or heme-containing systems, generating reactive radicals. This redox-dependent mechanism is central to the antimalarial activity of artemisinin derivatives and may also contribute to antimicrobial effects by promoting oxidative imbalance, membrane perturbation, and metabolic disruption in selected microorganisms [20,23].

Nile tilapia (*O. niloticus*) is highly perishable, and preserving its quality during storage is critical for reducing food waste and meeting nutritional demands. The aim of this study was to evaluate an experimental pre-harvest dietary approach based on supplementation with *A. annua* powder, artemisinin, and artemether, compounds with potential antioxidant-related and antimicrobial effects. The novelty of the present study lies in the dietary use of A. annua and its derivatives as a pre-harvest strategy for post-harvest fillet quality preservation, as well as in the comparison among the whole plant material, its major endoperoxide compound, and a semisynthetic derivative. These treatments were selected to compare the plant material, which contains several bioactive compounds with reported antioxidant and antimicrobial properties, with artemisinin, its major endoperoxide compound, and artemether, a semisynthetic derivative with distinct chemical characteristics. Their effects on the microbiological, physicochemical, and quality-related sensory attributes of fillets were evaluated over 30 days of refrigerated storage. We hypothesize that incorporating these bioactive compounds in the fish diet could influence shelf-life-related indicators of fillets by modulating oxidative stability, microbial development, or muscle biochemical characteristics before slaughter, thereby delaying quality deterioration during refrigerated storage.

## 2. Materials and Methods

### 2.1. Animals

The experimental procedures were approved by the Ethics Committee on the Use of Animals (CEUA), from UNESP/FCAV under protocol n° 1654/21. For the study 160 Nile tilapia from the same spawning were used, stored in 4 tanks (n = 40), with a capacity of 1000 L of water each, supplied with running water without chlorine, from an artesian well, with flow rate 1 L/min. After transport, fish were acclimated for 15 days so that the plasma cortisol concentration and osmolarity returned to baseline levels. In the first three days of acclimatization, they were subjected to baths in NaCl solution at a concentration of 6.0 g/L. The animals received commercial pelleted feed with 32% crude protein, constituting the basal diet. Fish were fed twice a day, fed at 8:00 a.m. and 6:00 p.m., corresponding to 2% of the biomass of the tanks. The water quality was determined twice a day (temperature and dissolved oxygen measured by the Oximeter (Xylem^®^, YSI model 55, Yellow Springs, OH, USA) and pH and electrical conductivity by the pH meter (YSI model 63, Yellow Springs, OH, USA) and their values remained within the range suitable for well-being within tropical fish [24] (dissolved oxygen = 4.09 ± 0.89 mg·L^−1^; temperature = 27.54 ± 2.05 °C; pH = 7.62 ± 0.54; and conductivity = 206.22 ± 97.57 μS/cm). Two daily cleaning by siphon method was applied to remove remained particles from the system.

### 2.2. Experimental Design

Fish were randomly distributed in 4 tanks (1000 L each), constituting the following treatments: T1: control (without treatment); T2: treated with *A. annua* (1%/kg of feed); T3: treated with artemisinin (9.6 mg/kg of feed) and T4: treated with artemether (9.6 mg/kg of feed). After 30 days of feeding, 320 fillets were collected and stored under refrigeration at 4 °C. Samples were analyzed at 0, 7, 15, and 30 days post-slaughter, 20 fillets per treatment were analyzed: 10 for microbiological counts (mesophilic and psychrotrophic bacteria, molds and yeasts, *Clostridium* sulfite-reducing, Enterobacteriaceae, Coagulase-positive staphylococci, and coliforms), and 10 for physicochemical (pH, color, lipid oxidation via TBARS), and sensory analysis.

### 2.3. Collection of Plant Material and Production of the Herbal Medicine

The *A. annua* variety from CPQBA-UNICAMP was cultivated in the city of Paulínia, State of São Paulo, Brazil (Lat. 22°48′02.38″ S, Long. 47°06′43.10″ W, altitude 612 m). Cultivation took place in the experimental field of the State University of Campinas, where the soil was classified as a typical Eutrophic Red Latosol. The plants were spaced 0.6 × 1.0 m apart [25]. Leaves were harvested before flowering, and only the upper part of each plant was selected for the current study. The leaves were then dried in a greenhouse with air circulation at an average temperature of 40 °C for 48 h [25]. The final raw material consisted of leaves, which were ground to a powder consistency.

### 2.4. Extraction and Purification of Artemisinin

*A. annua* (1 kg) was used to obtain artemisinin through ethanol extraction with mechanical agitation (3 times for 1.5 h) at room temperature. The extract was then filtered, and the solvent was removed under vacuum. The crude extract was purified by filtering the ethanolic crude extract (100 g dissolved in 500 mL of ethanol) with the aid of 10% activated charcoal (Carbomafra^®^, Curitiba, Brazil). The mixture was stirred at room temperature for half an hour, filtered again, and evaporated under vacuum at 40 °C [26]. The artemisinin-enriched extract was purified by column chromatography over silica gel 60 (70–230 mesh Merck^®^, Darmstadt, Germany) using different gradients of ethyl acetate-hexane. The separation process was monitored by thin-layer chromatography (TLC), and the isolated artemisinin was obtained as a pure compound, with purity confirmed by HPLC analysis using a commercial artemisinin standard (Sigma-Aldrich, St. Louis, MO, USA, CAS No. 63968-64-9) for comparison [27].

### 2.5. Preparation of Artemether

Artemether was synthesized from purified artemisinin through a two-step procedure, yielding a pure compound that was used in the experimental diets. First, artemisinin was chemically reduced to dihydroartemisinin, composed of α- and β-anomers, using sodium borohydride as reducing agent. The resulting dihydroartemisinin was then converted to artemether by acid-catalyzed methyl etherification in methanol containing a co-solvent. High-performance liquid chromatography (HPLC) was used for reaction monitoring and analytical evaluation of the products [28].

### 2.6. Experimental Diets

Commercial diets (NUTRIPISCIS^®^—Presence), containing 32% crude protein, 12% moisture, 60 g/kg ether extract (crude lipid fraction determined by ether extraction), 120 g/kg ash, 55 g/kg crude fiber, 25 g/kg calcium, 6 g/kg phosphorus, and 350 mg/kg vitamin C, were used as the base for the experimental diets. Daily, *A. annua* powder was incorporated at 1% of the feed, equivalent to 10 g/kg feed, while artemisinin and artemether were incorporated at 9.6 mg/kg feed. The different units were used because *A. annua* powder represents whole dried and ground plant material and was therefore expressed as a dietary inclusion percentage, while artemisinin and artemether are purified compounds and were expressed as mg/kg feed. The inclusion levels were based on previous studies with *Danio rerio* (zebrafish) conducted by our research group, which evaluated 1%, 3%, and 5% dried plant material. The 1% inclusion yielded the most favorable results, corresponding to approximately 9.6 mg of artemisinin/kg feed [19]. Therefore, artemisinin and artemether were included at 9.6 mg/kg feed to allow for comparison with the estimated artemisinin-equivalent amount provided by the 1% *A. annua* powder treatment. To prepare the experimental diets, *A. annua* powder, artemisinin, and artemether were mixed with soybean oil, comprising 2% of the total feed weight, to improve homogenization before incorporation into the feed. An equal amount of oil was added to the control diet to standardize fat content. All diets were freshly prepared daily in dark plastic containers with lids, homogenized using a vortex mixer, and stored at 8 °C until use. Fish were fed twice daily, at 8:00 a.m. and 6:00 p.m. No complete phytochemical profiling of the *A. annua* powder was performed in the present study.

### 2.7. Slaughter of Fish and Removal of Fillets

The stunning method performed was hypothermia, with purified water hyper chlorinated (5 ppm) and ice produced aseptically in the laboratory. The animals were taken in polystyrene thermal boxes covered by sterile plastic bags with a capacity of 16 L and kept in this volume for approximately 3 min, and every 10 animals were replaced with all material used during slaughter (ice, sterile plastic bag, knives, and others). After stunning the animals, brain stem decerebration was performed. The filleting process was performed by a single person using sterile materials in all groups (n = 10). A superficial cleaning of the fish was carried out to remove excess of blood and mucus with hyper chlorinated water at 5 ppm, followed by evisceration and filleting. The fillet was divided in the longitudinal direction, obtaining two parts (right and left) to be stored in sterile plastic bags and kept in controlled refrigeration at 4 °C. Standardization was carried out where the left side of the fish was used for microbiological analysis and the right side for physical-chemical analysis during the 30-day experimental period.

### 2.8. Microbiological Analysis

#### 2.8.1. Preparation of Dilutions

Twenty-five grams of each sample collected from different points (surface and internal portions) were transferred into a sterile stomacher bag, and 225 mL of sterile 0.1% saline-peptone water (HiMedia Laboratories^®^, Kennett Square, PA, USA) was added. The mixture was homogenized for 3 min using a Stomacher NT-120 homogenizer (Novatecnica, Piracicaba, Brazil) to obtain the initial 10^−1^ dilution. Subsequently, serial ten-fold dilutions were prepared by transferring 1 mL of the previous dilution into tubes containing 9 mL of sterile 0.1% saline-peptone water to obtain the 10^−2^, 10^−3^, and 10^−4^ dilutions.

#### 2.8.2. Counts of Aerobic and Facultative Anaerobic Mesophilic Microorganisms

This analysis was performed following the APHA 08:2015 methodology [29]. The chosen plating technique was the pour plate method. For each dilution, 1 mL was transferred into sterile Petri dishes, followed by the addition of 20 mL of Plate Count Agar (PCA, Kasvi^®^. Pinhais/PR, Brazil), previously melted and maintained in a water bath at 46–48 °C. The plates were gently homogenized and incubated at 36 °C for 48 h. After incubation, the colonies were counted. The number of colonies was then multiplied by the respective dilution factor, and the results were expressed as colony-forming units per gram (CFU/g).

#### 2.8.3. Counts of Aerobic and Anaerobic Facultative Psychrotrophic Microorganisms

This analysis was conducted following the APHA 13.61:2015 method [30]. The spread plate method was used for the enumeration of psychrotrophic microorganisms. Approximately 20 mL of Plate Count Agar (PCA, Kasvi^®^, Castelfidardo, Italy), previously melted and maintained in a water bath at 46–48 °C, was poured into sterile Petri dishes. After solidification at room temperature, 0.1 mL of each dilution was surface-inoculated. The plates were then incubated at 7 °C for 10 days. Colony counts were multiplied by the corresponding dilution factor and further multiplied by 10, since only 0.1 mL of each dilution was used. Results were expressed as colony-forming units per gram (CFU/g).

#### 2.8.4. Counts of Total and Thermotolerant Coliforms

This analysis was performed according to the APHA 9:2015 method [31]. The Most Probable Number (MPN) technique was used to estimate counts of specific microorganisms, such as total and thermotolerant coliforms. For the presumptive test, serial dilutions (10^−1^, 10^−2^, and 10^−3^) were prepared in triplicate using sterile tubes containing Lauryl Sulfate Tryptose (LST) broth (Himedia Laboratories^®^, Sao Paulo, Brazil) with inverted Durham tubes. The tubes were incubated at 36 °C for 48 h. Gas production in the Durham tubes was considered a positive result. For the confirmation of total coliforms, a loopful from positive presumptive tubes was transferred to tubes containing Brilliant Green Lactose Bile (BGLB) broth (Kasvi^®^) and incubated at 36 °C for 48 h. For thermotolerant coliforms, the EC broth (Kasvi^®^) was used, and incubation was carried out in a water bath with circulation at 45 °C for 48 h. The presence of gas in the Durham tubes in the confirmatory tests indicated a positive result. Final counts were determined using the MPN method [32,33].

#### 2.8.5. Mold and Yeast Counts

This analysis was performed according to APHA 21:2015 [34]. From the serial dilutions, the pour plate method was employed, in which 1 mL of each sample was transferred to sterile Petri dishes, followed by the addition of 20 mL of Potato Dextrose Agar (Merck^®^), previously adjusted to pH 3.5 ± 1. The plates were gently homogenized and incubated without inversion at 25 ± 1 °C for 7 days. After the incubation period, colonies were counted. The number of colonies was multiplied by the corresponding dilution factor, and the results were expressed as colony-forming units per gram (CFU/g).

#### 2.8.6. *Clostridium* Sulfite Reducing Count

This analysis was performed according to APHA 33.72:2015 [35]. From the sample dilutions, the pour plate method was chosen. One milliliter (1 mL) of each dilution was inoculated into sterile Petri dishes following an adapted methodology, and 20 mL of SPS (Sulfite Polymyxin Sulfadiazine) Agar (Merck^®^) was added. The plates were gently homogenized and allowed to solidify. Subsequently, a second overlay (10 mL) of the same medium was added, solidified, and incubated without inversion in an anaerobic jar at 36 ± 1 °C for 24 h. After the incubation period, typical colonies of sulfite-reducing *Clostridium* were counted. The number of colonies was multiplied by the corresponding dilution factor, and results were expressed as colony-forming units per gram (CFU/g).

#### 2.8.7. Total Enterobacteriaceae Counts

This analysis was performed according to APHA 9.62:2015 [31]. Similarly, 1 mL of each dilution was transferred to sterile Petri dishes using the pour plate technique. Then, 20 mL of MacConkey Agar (Himedia Laboratories^®^), previously melted and maintained in a water bath at 46–48 °C, was added according to an adapted methodology. The plates were gently mixed and allowed to solidify on a flat surface. Afterward, a second overlay (10 mL) of the same medium was added, allowed to solidify, and the plates were incubated at 36 ± 1 °C for 24 h. After this period, colonies were counted. The number of colonies was multiplied by the appropriate dilution factor, and the result was expressed as colony-forming units per gram (CFU/g).

#### 2.8.8. Total Count Coagulase-Positive Staphylococci

This analysis was performed according to the APHA 39.63:2015 method [36]. To count *Staphylococcus* spp. 0.1 mL of each dilution was added per surface, spread plate, Baird-Parker Agar (Kasvi^®^, Castelfidardo, Italy), supplemented with egg yolk solution with potassium tellurite (Dinamica^®^, Indaiatuba, Brazil). With the help of a “hockey” stick, the inoculum was carefully spread over the entire surface of the medium until it was completely absorbed. The plates were incubated inverted at 36 ± 1 °C for 48 h. The number of colonies counted was multiplied by the corresponding dilution factor, and multiplied by 10, and the result obtained was expressed in colony forming units per gram (CFU/g). For the coagulase test, 3–5 typical and atypical colonies were selected and seeded in BHI broth (Kasvi^®^, Italy), and incubated at 36 ± 1 °C for 24 h. After growth, 0.3 mL of plasma was transferred to sterile tubes containing 0.3 mL of rabbit plasma (Newprov^®^, Pinhais, Brazil), incubated at 36 ± 1 °C for 6 h, and the presence of clots was verified. Gram staining was performed by preparing the smear and staining using the Gram method. For the Catalase test, an aliquot was transferred to a glass slide containing a drop of 3% hydrogen peroxide, the inoculum was homogenized and the reaction was observed, and the non-formation of bubbles indicates a negative test, as the formation of bubbles indicates a positive test for catalase [32].

### 2.9. Physicochemical Analysis

#### 2.9.1. Determination of pH

The pH was determined in duplicate in the epaxial muscle, using a digital pH meter (Testo^®^, model 205. West Chester, PA, USA), equipped with a penetration electrode, through direct insertion.

#### 2.9.2. Colorimetry

It was determined through CR-400 Chroma Meter colorimeter (Konica Minolta Sensing Americas, Inc., Ramsey, NJ, USA) which uses the CIELAB system (L, a* and b*). Parameters such as luminosity (L*), red intensity (a*) and yellow intensity (b*) of the epaxial muscle were evaluated. The evaluation was performed at three different points in each part of the muscle, to obtain an average of the values. The color intensity is expressed by a chroma value (C*ab), while matrix (H0ab) corresponds to the name of the color found in its pure state in the spectrum. These values were calculated using the formulas: C*ab = (a*2 + b*2)1/2 according to [1], and hue of color is an angular measure where 0° indicates a red hue, 90° denotes a yellow hue, 180° green and 270° blue, being determined by the equation, H0ab = arctan (b*/a*).

#### 2.9.3. Lipid Oxidation (TBARS)

Fillet samples of 5 g were ground in Falcon tubes for lipid oxidation analysis, following the methodology described by Pikul [37]. Then, 25 mL of 7.5% trichloroacetic acid (TCA; Synth^®^, Diadema, Brazil) was added to each tube, one tube at a time, to prevent excessive sample dehydration during acid addition. The mixture was homogenized using an Ultra-Turrax homogenizer for 1 min and then filtered into another Falcon tube. All samples were analyzed in triplicate, and 5 mL of 0.01 M thiobarbituric acid (TBA; Synth^®^, Brazil) was added to 5 mL of the extract. Then the mixture was heated in a water bath at 100 °C for 40 min, with the test tubes covered with glass marbles. For TBARS quantification, a standard curve was constructed using nine dilution points of 1,1,3,3-tetraethoxypropane (TEP) as the malondialdehyde (MDA) precursor. The standard curve showed a coefficient of determination of R^2^ = 0.98. The determination of TBARS, expressed as mg of malondialdehyde/kg of sample, was performed using spectrophotometry (Shimadzu UV-1800, Tokyo, Japan) at 532 nm.

### 2.10. Sensory Analysis

The sensory quality of tilapia fillets was evaluated as a supportive descriptive assessment in each sampling period by six trained panelists. This evaluation was not intended to represent a Quantitative Descriptive Analysis (QDA) or a consumer acceptance test. The assessment was performed on raw fillets and did not involve tasting or ingestion of the samples. Fish samples (150 g) from the dorsal region were individually presented to the panelists in individual booths under controlled conditions of light, temperature, and humidity. Panelists were asked to rate the characteristics of brightness, texture, appearance, and odor using a scale of 0 to 10, with 10 being the best condition and 0 the worst. Texture was evaluated based on visual and tactile perception of firmness, elasticity, surface integrity, and evidence of muscle softening or loss of structure. Odor was evaluated through controlled olfactory assessment immediately after sample presentation, considering the presence of characteristic fresh fish odor and the intensity of spoilage-related off-odors. The following quality categories were scored: excellent quality (10.00–9.00); optimal quality (8.00–7.00); good quality (6.00–5.00); regular quality (4.00–3.00); bad quality (2.00–1.00); poor quality (1.00–0.00) [1].

### 2.11. Statistical Analysis

The experimental data were subjected to analysis of variance through a completely randomized design in a factorial scheme with a “Split Plot Design” (4 treatments X 4 periods), using the SAS statistical package, using the PROC GLM procedure, version 9.3 [38]. Multiple comparisons were measured using the Tukey test at the 95% confidence level. All variables obtained in this study were analyzed using Principal Component Analysis (PCA) with the PAST 5.0 software (Paleontological Statistics). Following the initial PCA, only variables with loadings greater than ±0.80 (80%) in at least one of the principal components (PC1, PC2, or PC3) were selected for a second, refined PCA. The PCA was used as a parameter to perform Spearman correlations (SAS 9.3).

## 3. Results

### 3.1. Microbiological Analysis

**Figure 1:**
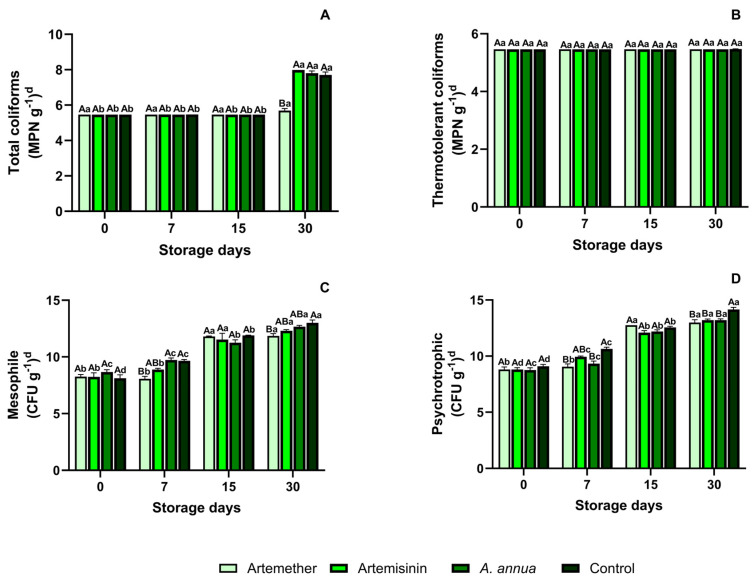
Microbiological analyses of tilapia fillets from the artemether, artemisinin, *Artemisia annua* powder, and control groups. (**A**) Total coliforms; (**B**) thermotolerant coliforms; (**C**) mesophilic microorganisms; (**D**) psychrotrophic microorganisms. Data are presented as mean ± standard error of the mean (SEM) (n = 10 fish). Means followed by the same letter do not differ significantly by Tukey’s test (*p* < 0.05). Uppercase letters compare treatments within each experimental period, while lowercase letters evaluate the evolution of each treatment over the experimental periods. Sampling periods: 0, 7, 15, and 30 days post-slaughter (DPS).

The MPN values for thermotolerant coliforms did not differ significantly (*p* ≥ 0.05) among treatments (Figure 1B). However, total coliform counts were significantly lower in fillets from fish supplemented with artemether after 30 days of refrigerated storage (Figure 1A).

The count of mesophilic (Figure 1C) and psychrotrophic (Figure 1D) microorganisms at 7 and 30 DPS was significantly lower (*p* < 0.05) in fillets from treated fish, with emphasis on the group treated with artemether, when compared with fillets from the control group. All treatments showed a significant increase (*p* < 0.05) in the count of mesophilic and psychrotrophic microorganisms over the 30 days of refrigerated storage; however, this increase was more pronounced in fillets from the control group (Figure 1C,D).

**Figure 2:**
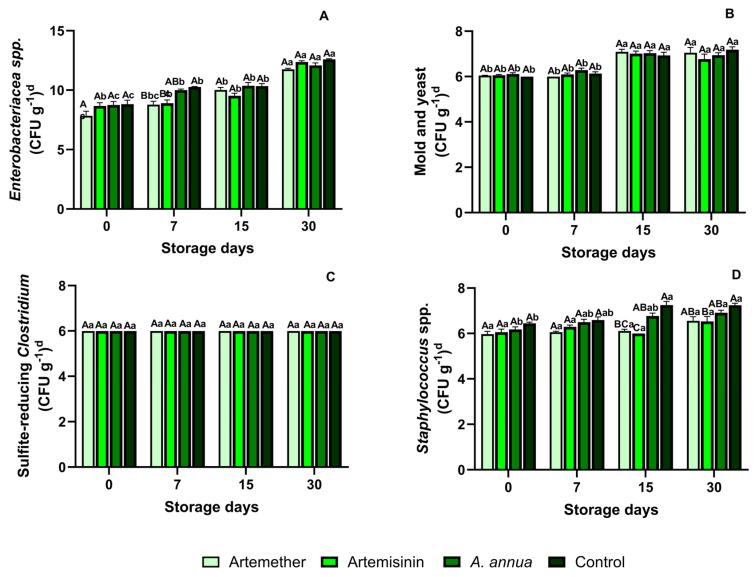
Microbiological analyses of tilapia fillets from the artemether, artemisinin, *Artemisia annua* powder, and control groups. (**A**) Enterobacteriaceae; (**B**) molds and yeasts; (**C**) sulfite-reducing *Clostridium*; (**D**) coagulase-positive staphylococci. Data are presented as mean ± standard error of the mean (SEM) (n = 10 fish). Means followed by the same letter do not differ significantly by Tukey’s test (*p* < 0.05). Uppercase letters compare treatments within each experimental period, while lowercase letters evaluate the evolution of each treatment over the experimental periods. Sampling periods: 0, 7, 15, and 30 days post-slaughter (DPS).

A significant decrease in the count of Enterobacteriaceae colonies was observed in fillets from fish treated with artemether and artemisinin after 7 days of refrigerated storage, compared with fillets from the control group. However, over time, there was an increase in all groups kept under refrigeration (Figure 2A).

In the count of molds and yeasts, no significant differences were observed among treatments (*p* ≥ 0.05). However, counts increased after 15 days of refrigerated storage in all groups evaluated (Figure 2B). No growth of sulfite-reducing *Clostridium* was detected in any treatment throughout the storage period; therefore, no significant differences were observed among treatments (Figure 2C).

The count of Coagulase-positive staphylococci was significantly higher (*p* < 0.05) at 15 and 30 days of shelf life in fillets belonging to the control fish group, compared to fillets from treated fish (Figure 2D). Throughout the refrigeration period, an increase in Coagulase-positive staphylococci counts was observed in fish fillets from the control group and those treated with *A. annua*.

### 3.2. Physicochemical Analysis

**Figure 3:**
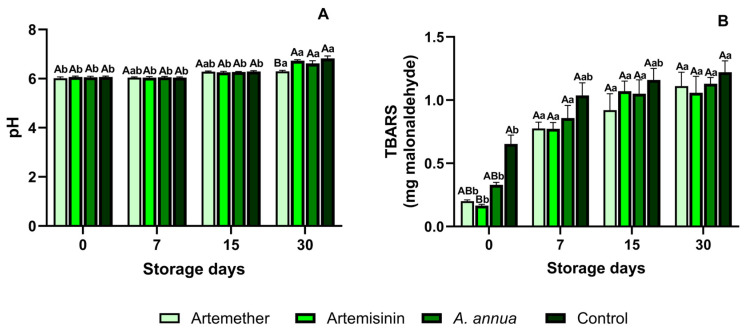
Physicochemical analyses of pH and TBARS of tilapia fillets from the artemether, artemisinin, *Artemisia annua* powder, and control groups. (**A**) pH; (**B**) TBARS. Data are presented as mean ± standard error of the mean (SEM) (n = 10 fish). Means followed by the same letter do not differ significantly by Tukey’s test (*p* < 0.05). Uppercase letters compare treatments within each experimental period, while lowercase letters evaluate the evolution of each treatment over the experimental periods. Sampling periods: 0, 7, 15, and 30 days post-slaughter (DPS).

Measuring the pH of the fillets demonstrated that tilapia that were treated with artemether showed a significant reduction *(p* < 0.05) after 30 days, compared to the other groups. (Figure 3A). Over time, it was observed that the animals remained stable for up to 15 days of refrigeration and after 30 days they showed an increase in pH values in all groups (Figure 3A).

Analysis of TBARS (Figure 3B) revealed that animals in the control group presented significantly higher values (*p* < 0.05) on day 0 compared to treated animals (Figure 3A). As expected, there was an increase in TBARS values in all groups over time.

#### Colorimetry

**Figure 4:**
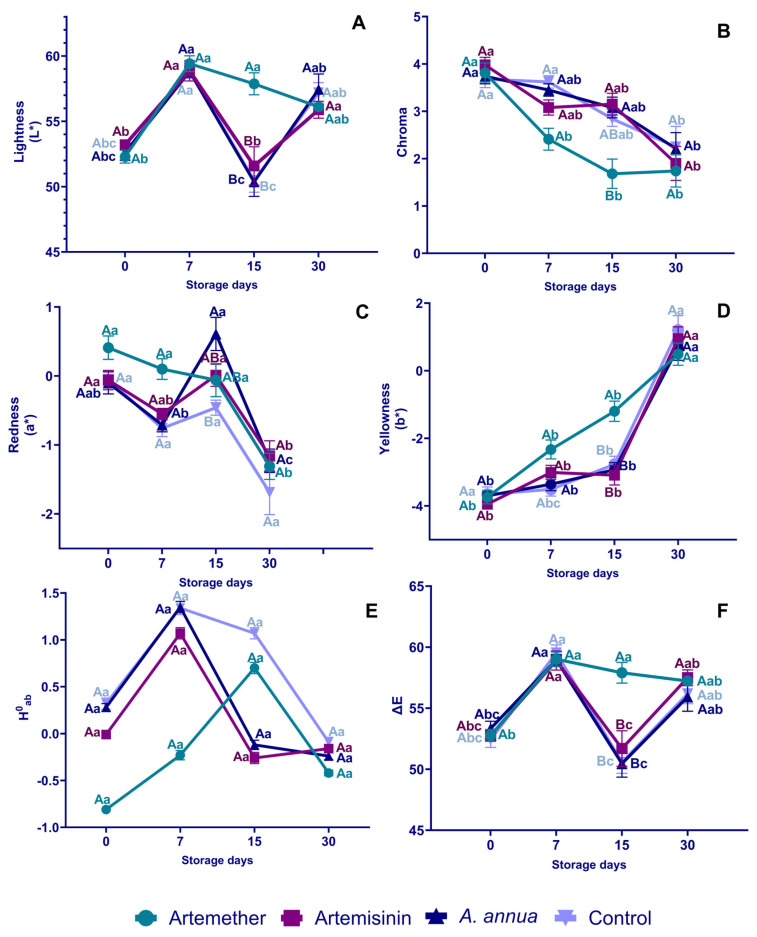
Colorimetric evaluation of tilapia fillets from the artemether, artemisinin, *Artemisia annua* powder, and control groups. (**A**) Lightness (L*); (**B**) chroma (C*ab); (**C**) red–green coordinate (a*); (**D**) blue–yellow coordinate (b*); (**E**) hue angle (h°ab); (**F**) total color difference (ΔE). Data are presented as mean ± standard error of the mean (SEM) (n = 10 fish). Means followed by the same letter do not differ significantly by Tukey’s test (*p* < 0.05). Uppercase letters compare treatments within each experimental period, while lowercase letters evaluate the evolution of each treatment over the experimental periods. Sampling periods: 0, 7, 15, and 30 days post-slaughter (DPS).

In the colorimetric analysis carried out on the 15th day of storage, it was observed that animals treated with artemether presented higher luminosity values (0 = black and 100 = white), that is, closer to white, compared to the other treatments (Figure 4A).

Analysis of the intensity from red (a*+) to green (a*−) (Figure 4C) revealed that the fillets from the control group showed a significant difference at 15 days, becoming colors close to the shade of green (−0.46), while the animals treated with *A. annua* had colors close to Red (0.61).

In the colorimetric evaluation of color intensity, ranging from yellow (positive b*) to blue (negative b*), it was observed that fillets treated with artemether presented lower negative b* values compared to the other treatments, (Figure 4D). Furthermore, it was noted that at the maximum refrigeration period for the fillets (30DPS), all the files had shades close to green (a*−) and yellow (b*+).

Tilapia fillets treated with artemether showed a reduction in chroma saturation index (c*) values compared to other treatments at 15 DPS (Figure 4B). Furthermore, it showed a significant gradual decrease (*p* < 0.05) for all treatments up to 30 DPS. The analysis of the total color difference expressed in Delta E (∆E) at 15 days of storage revealed that the values were significantly higher (*p* < 0.05) for fillets from animals treated with artemether compared to the other groups (Figure 4F). Meanwhile, the Hue inclination angle (H*ab) values did not show significant differences. This indicates that, despite variations in terms of luminosity (L*) and color intensity (a* and b*), the basic color tone of the fillets remained unchanged (Figure 4E).

### 3.3. Sensory Quality Assessment

**Figure 5:**
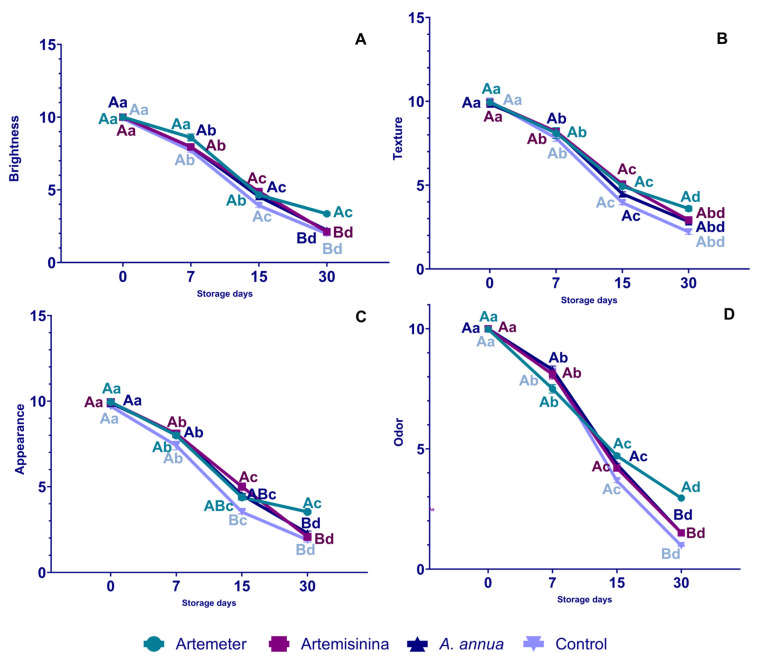
Sensory quality assessment of tilapia fillets from the artemether, artemisinin, *Artemisia annua* powder, and control groups. (**A**) Brightness; (**B**) texture; (**C**) appearance; (**D**) odor. Data are presented as mean ± standard error of the mean (SEM) from fillet samples (n = 10 fish) evaluated by six trained panelists. Different letters indicate significant differences by Tukey’s test (*p* < 0.05). Uppercase letters compare treatments within each experimental period, while lowercase letters indicate changes within each treatment over the experimental periods. Sampling periods: 0, 7, 15, and 30 days post-slaughter (DPS).

In the sensory quality assessment, considering brightness, texture, appearance, and odor, tilapia fillets from fish subjected to artemether treatment generally presented better classifications (Figure 5). In contrast, fillets from the control group, which did not receive any dietary supplementation, presented the lowest classifications.

The brightness scores of the fillets evaluated in the sensory quality assessment showed a significant difference (*p* < 0.05) at 30 days of refrigerated storage, with fillets from fish treated with artemether exhibiting higher values than those from the other groups (Figure 5A). During storage, fillets showed a significant decrease in brightness from day 7; however, in fillets from fish fed artemether, this decrease was only observed after 15 DPS.

For texture and odor, fillets from the control group presented lower scores (*p* < 0.05) than fillets from supplemented fish at 30 DPS (Figure 5B,D). Over the refrigerated storage period, a significant worsening of texture and odor was observed in all treatments; however, this deterioration was less pronounced in fillets from fish treated with artemether.

**Figure 6:**
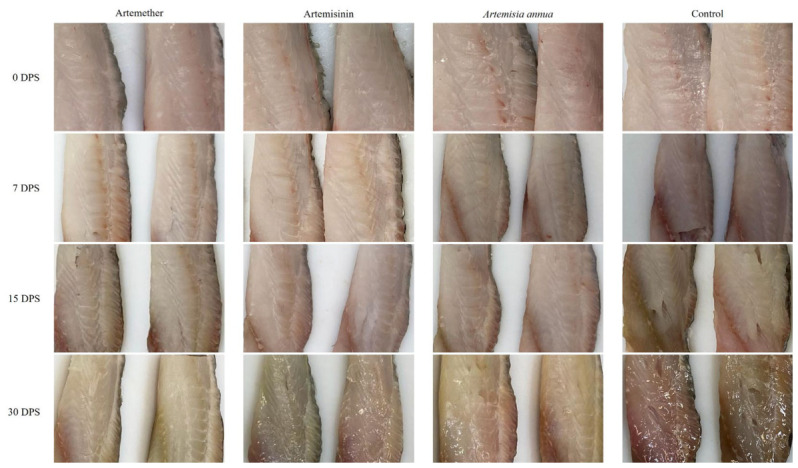
Representative photographs of Nile tilapia fillets from the artemether, artemisinin, *Artemisia annua* powder, and control groups during refrigerated storage at 0, 7, 15, and 30 days post-slaughter (DPS). The images are presented as a visual complement to the appearance evaluation. Objective color interpretation was based primarily on instrumental colorimetric measurements.

For appearance, fillets from fish receiving dietary supplementation showed better scores (*p* < 0.05) at 15 DPS for artemisinin and at 30 DPS for artemether when compared with control fillets (Figure 5). Representative photographs of the fillets at each storage time are presented in Figure 6 as a visual complement to the appearance evaluation. Throughout storage, a gradual reduction (*p* < 0.05) in appearance scores was observed in all treatments. At 30 DPS, fillets from fish fed artemether remained in better condition and maintained values close to those observed at 15 DPS.

### 3.4. Principal Component Analysis (PCA)

**Figure 7:**
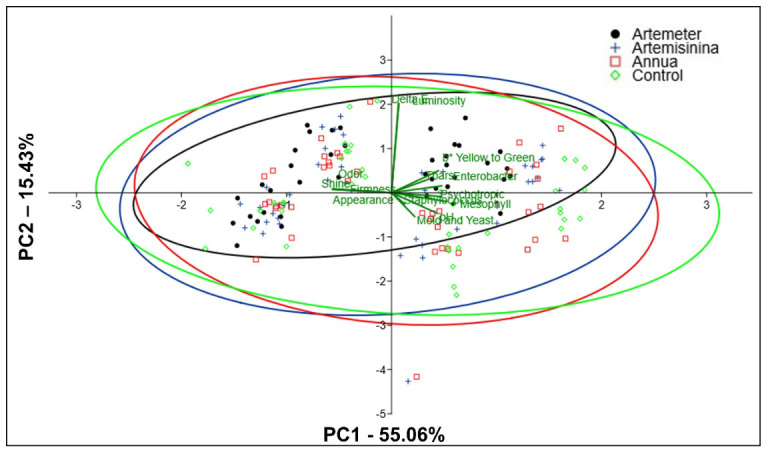
Principal Component Analysis (PCA) of variables obtained after Nile tilapia treatment with *A. annua*, Artemisinin, Artemether and control (untreated). The relationships among variables are represented through principal components, where the direction and length of vectors indicate the strength and direction of loadings.

Principal component analysis (PCA) helps to understand the interactions (correlation) between the variables studied. A homogeneous distribution of the experimental groups was observed in the PCA scatterplot (Figure 7). The principal components obtained explained 77.61% of the total variance among variables (PC1: 55.06%; PC2: 15.43%; PC3: 7.12%). This cumulative variance indicates that the main sources of variation were represented by the first three components, although part of the variability remained distributed across additional components. This distribution may be related to the multifactorial nature of the dataset, which included microbiological, physicochemical, lipid oxidation, colorimetric, and sensory quality parameters. Therefore, PCA was used as an exploratory multivariate approach to visualize the main relationships among variables, treatments, and storage periods.

**Figure 8:**
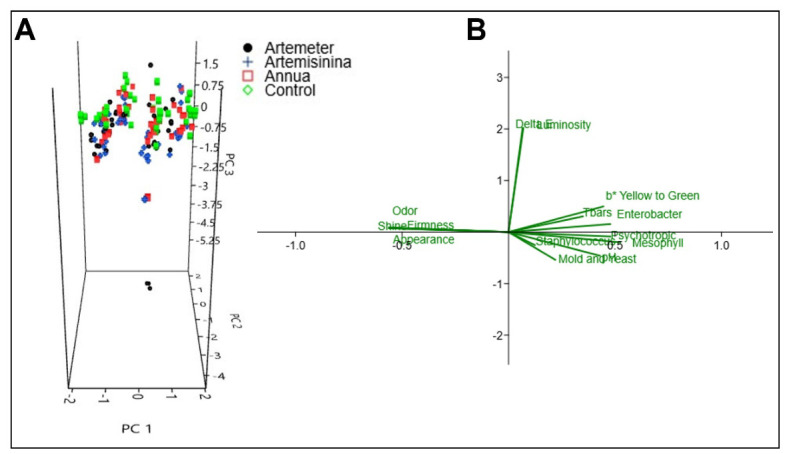
Distribution of variables across principal components 1, 2, and 3. (**A**) The 3D spatial representation of variable loadings across the three components. (**B**) Vector length and direction indicate the magnitude and sign (positive or negative) of loadings in the PCA.

The spatial distribution of the data and the orientation of the vectors are presented in Figure 8. Variables such as Delta E, luminosity, b*, TBARS, Enterobacteriaceae, psychrotrophs, mesophiles, *Staphylococcus*, molds, and yeasts exhibited positive loadings, as they are located in the quadrant where both X and Y axes are positive (PC1, PC2, and PC3). In contrast, brightness, firmness, appearance, and odor showed negative loadings in PC1.

**Figure 9:**
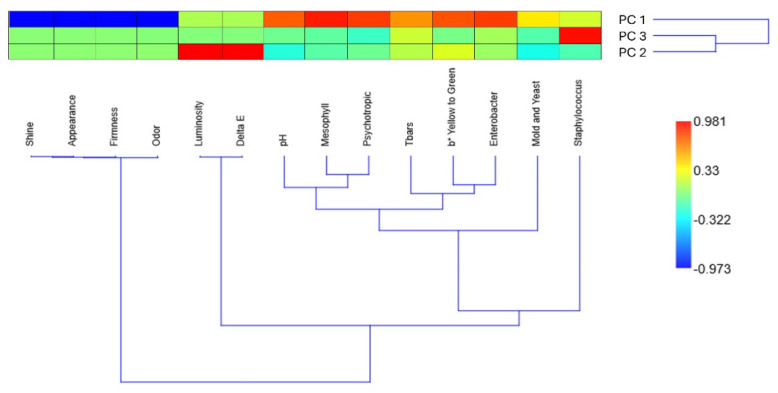
Heat map of variable loadings in PCA. Higher positive loadings in PC1, PC2, and PC3 are shown in red; negative loadings are shown in blue. Cluster relationships between variables are indicated below the heat map.

Based on cluster analysis and the heat map (Figure 9), it is possible to assess the correlation among the variables across the four experimental groups. Variables with positive loadings in PC1, such as Enterobacteriaceae, mesophiles, and psychrotrophs, showed significant positive correlations (ρ ≥ 0.70; *p* < 0.05) within all four groups. Similarly, variables with negative PC1 loadings, luminosity, firmness, appearance, and odor, were also positively correlated among themselves (ρ ≥ 0.90; *p* < 0.05). Conversely, when comparing positively loaded variables (e.g., Enterobacteriaceae, mesophiles, psychrotrophs) with negatively loaded variables (e.g., luminosity, firmness, appearance, odor) in PC1, a significant negative correlation was observed (ρ ≤ −0.70; *p* < 0.05). Luminosity and Delta E presented high loading in PCA2 with positive correlation (Figure 9). Coagulase-positive staphylococci were the only variable with a high loading in PC3, showing a positive correlation.

Correlation analysis between microorganism counts and pH values revealed that control animals, treated with *A. annua*, and Artemisinin showed a significant positive correlation (*p* < 0.05) of 71.0, 60.8, and 64.1% between increases in coliform microorganism counts and elevations in pH values, respectively (Table 1). Fillets from control fish and those treated with *A. annua* powder showed a positive correlation (*p* < 0.05) of 57.5 and 43.8% between increases in pH values and coagulase-positive staphylococci counts during storage (Table 1). The mesophilic and total coliform counts showed a positive correlation (*p* < 0.05) of 73.3, 76.2, and 66.4% in tilapia fillets from the control group, treated with *A. annua* powder. and artemisinin, respectively (Table 1). Increases in coagulase-positive staphylococci counts were positively correlated (*p* < 0.05) with increases in mesophilic counts (63.1% and 66.7%) and psychrotrophic counts (58.1% and 50.5%) in fillets of control and *A. annua*-treated fish, respectively (Table 1).

Correlation analysis between TBARS values and colorimetric parameters revealed that fillets artemisinin- and artemether-treated tilapia showed a significant (*p* < 0.05) positive correlation with Lightness (L*) (36.8 and 65.8%, respectively) and total color difference (∆E) (35.4 and 65.5%, respectively), as well as a significant (*p* < 0.05) negative correlation with Chroma (50.4 and 71.0%, respectively) (Table 2). Fillets from fish treated with *A. annua* powder showed a negative correlation (*p* < 0.05) of 50.5% between the increase in TBARS values and Chroma during storage (Table 2).

## 4. Discussion

Freshness and quality were evaluated using microbiological, physicochemical, and supportive sensory quality analyses. The Most Probable Number (MPN) method for coliforms is a widely used indicator of hygienic–sanitary conditions in foods [31,32]. Across 30 days at 4 °C, tilapia fillets showed low thermotolerant coliform counts, likely reflecting controlled rearing conditions, careful sampling, and processing. *Escherichia coli* is not part of the natural fish microbiota, and its detection is usually linked to contamination along the production chain and environmental sources [31]. In this study, good water quality and rigorous slaughter/handling protocols likely contributed to the low counts, supporting the role of processing hygiene in refrigerated fillet quality. Notably, total coliforms decreased in the artemether-supplemented group, suggesting a possible contribution of dietary supplementation to improved microbial stability. This response is relevant because microbial development during refrigerated storage is one of the main drivers of fish quality deterioration and shelf-life reduction.

Members of the Enterobacteriaceae family are commonly used as indicators of contamination in aquaculture and fish products, as they comprise abundant potentially pathogenic bacteria and include opportunistic fish pathogens [39]. Elevated counts of Enterobacteriaceae are often used in food safety assessments to indicate inadequate hygiene practices, which may increase the risk of pathogen exposure and reduce product shelf life [40,41]. Here, enumeration of Enterobacteriaceae revealed a significant increase in the control group after 7 days of refrigeration compared with supplemented groups. Similar to the pattern observed for total coliforms, these findings suggest that dietary supplementation may have contributed to delayed microbial proliferation or improved microbial stability, rather than indicating a direct post-harvest antimicrobial effect on the fillet surface. Nevertheless, microbial counts remained relatively stable over subsequent storage periods in all treatments, indicating that the dietary effect was more evident during specific phases of refrigerated storage.

Fish and fish products provide favorable substrates for the growth of spoilage microorganisms [11,42]. Across refrigerated storage, tilapia fillets showed progressive increases in mesophilic and psychrotrophic counts; however, artemether supplementation significantly reduced bacterial loads, with the control group exhibiting higher counts than treated fish at 7 and 30 days. These results suggest that the dietary treatments, especially artemether, may have contributed to improved microbial stability of the fillets during refrigerated storage. Similar responses have been reported in studies evaluating pre-harvest nutritional approaches in fish, including dietary astaxanthin and green microalgae supplementation in Nile tilapia, which improved microbial and quality-related indicators of refrigerated fillets [43,44]. Although these studies are particularly close to the present experimental model, broader evidence also indicates that dietary antioxidants and plant-derived feed additives can modulate oxidative status, immune responses, and product quality in aquaculture species [45]. Beyond spoilage, mesophilic bacteria are relevant to food safety because most human pathogens are mesophiles, growing optimally near 37 °C [32].

On the other hand, psychrotrophic microorganisms are capable of growing under refrigeration, contributing to food spoilage and posing potential health risks to consumers [42]. In refrigerated products, psychrotrophic bacteria are known to secrete heat-stable lipases and proteases, which can adversely affect food quality even after thermal processing [42]. Under the conditions evaluated here, the most favorable microbiological outcomes were observed in fish fed with artemether, indicating its potential role in delaying spoilage-related bacterial growth during refrigerated storage. This effect may be especially important for refrigerated fish, because psychrotrophic bacteria are among the main contributors to quality loss under low-temperature conditions.

Food spoilage results from microbial activity, including bacteria, yeasts, and molds, and chemical changes that can compromise product acceptability [40]. Yeasts are particularly relevant in fish because they can proliferate under refrigeration (4 °C) [46,47]. In the evaluated fillets, molds and yeasts were not detected before day 15, appeared thereafter in all treatments, and remained at very low levels through 30 days, with no differences among groups. Similarly, sulfite-reducing microorganisms did not differ between treatments. Because sulfite-reducing microorganisms may be associated with environmental contamination and hygiene failures in the fish production or handling chain, their presence is usually linked to contamination at the production site or along the handling chain, including water, ice, equipment, and utensils [48,49] and are not typical components of the fish microbiota, their presence is usually linked to contamination at the production site or along the handling chain, including ice, equipment, and utensils [50]. Consistent with prior storage studies, counts were low or undetectable [51,52,53], and under the present conditions no clear effect of dietary supplementation on this group could be established.

Coagulase-positive staphylococci counts were significantly higher after 15 and 30 days of shelf life in fillets from the control group compared with fillets from supplemented fish. Throughout the refrigeration period, an increase in coagulase-positive staphylococci counts was observed in fillets from the control group and from fish supplemented with *A. annua* powder. *Staphylococcus aureus* is a common foodborne pathogen capable of causing a variety of human infections [54]. Previous studies have reported inhibitory effects of *Artemisia* species and their bioactive compounds against Gram-positive bacteria, including *S. aureus* [20,55]. Although those studies do not reproduce the dietary approach used here, they support the biological plausibility that compounds present in *Artemisia* may be associated with antimicrobial-related responses. In the evaluated fillets, coagulase-positive staphylococci counts were predominantly higher in the control group, suggesting that dietary supplementation may have contributed to reduced bacterial growth during storage. This response may be associated with antimicrobial-related properties of bioactive compounds present in *A. annua* and with indirect pre-harvest effects of dietary supplementation on muscle condition, microbial ecology, or microbial development after slaughter.

The antimicrobial and antioxidant-related effects observed here should be interpreted together. Artemisinin and artemether should not be described only as classical antioxidants, because their endoperoxide bridge can be activated in the presence of Fe^2+^ or heme-containing systems, generating reactive radicals, a mechanism classically associated with the antimalarial activity of artemisinin derivatives [23]. This redox-dependent activity has also been discussed in relation to the antimicrobial potential of Artemisia, artemisinin, and its derivatives [20]. In addition, other bioactive compounds present in *A. annua*, such as flavonoids, phenolic acids, terpenoids, and volatile constituents, may contribute to both antioxidant and antimicrobial responses [13,14]. Therefore, the lower TBARS values and reduced counts of selected microbial groups in fillets from supplemented fish may be explained, at least in part, by combined effects on secondary lipid oxidation products and spoilage-associated microbial development, rather than by a single direct antioxidant mechanism.

Oxidative processes are among the main factors responsible for quality loss during refrigerated storage of fish products. Fish muscle is particularly susceptible to lipid oxidation because of its high content of polyunsaturated fatty acids, and low-temperature storage only partially slows oxidative reactions [56,57]. Reactive oxygen species (ROS), such as oxygen-centered radicals (O•), hydroxyl radicals (•OH), and superoxide anions (O_2_•^−^), can react with lipids and proteins, accelerating oxidative spoilage [56,57,58]. Thiobarbituric acid reactive substances (TBARSs) quantify secondary lipid-oxidation products, mainly aldehydes and other carbonyl compounds derived from polyunsaturated fatty acids, which are closely associated with rancid off-flavors and off-odors in fish products [56,57]. During refrigerated storage, TBARS values increased, indicating a progressive increase in secondary lipid oxidation products, as estimated by the TBARS assay.

Interestingly, differences among treatments were already observed at day 0, immediately after slaughter. Since this sampling point occurred after the 30-day feeding period, these initial differences may reflect the influence of dietary supplementation on the oxidative condition of the muscle before storage began. Dietary antioxidants and bioactive compounds may modulate lipid stability, antioxidant defenses, and muscle biochemical characteristics, thereby affecting the susceptibility of fillets to oxidation after slaughter [43,56]. Thus, the lower initial TBARS values observed in fillets from supplemented fish may reflect a pre-harvest effect of dietary supplementation on muscle oxidative status, rather than a post-harvest surface effect. In fish, dietary supplementation with vitamin E, α-tocopheryl acetate, essential oils, and plant-derived feed additives has been associated with improved oxidative stability and delayed quality deterioration during refrigerated or ice storage [45,59,60,61,62,63]. In addition, *A. annua* contains phenolic compounds, flavonoids, terpenoids, and sesquiterpene lactones that have been associated with antioxidant-related and antimicrobial responses [12,13,17]. In this context, the effects on fillet oxidation may depend on bioavailability, possible tissue deposition, or indirect modulation of muscle biochemical characteristics before slaughter. Nevertheless, because residue deposition and direct antioxidant activity were not evaluated, the lower TBARS values should be interpreted as evidence of improved fillet oxidative stability rather than as definitive evidence of direct antioxidant activity in the fillets. Although TBARS is widely used as an indicator of lipid oxidation and oxidative quality deterioration in fish products [56], it is not generally established as a direct regulatory limit for refrigerated fish fillets; therefore, these results should not be interpreted as direct evidence of regulatory compliance or commercial shelf-life extension [49].

pH is a relevant indicator of fish freshness and storage stability and is closely associated with microbial development, chemical reactions, and biochemical deterioration that contribute to the deterioration of food products, influencing sensory characteristics such as odor, color, and texture [3]. During refrigerated storage, spoilage microorganisms can degrade low-molecular-weight nitrogenous compounds, including amino acids and other substrates present in fish muscle, leading to the accumulation of alkaline metabolites and, consequently, increased pH values [64,65]. This scenario was observed in our study, where tilapia fillets showed a significant increase in pH values after 30 days of refrigeration, indicating greater deterioration in the fish fillets.

Studies that evaluated the quality of Nile tilapia fillets using pH as one of the indicators demonstrated that pH changes are closely associated with microbial development, biochemical deterioration, and loss of freshness during refrigerated storage [64,65,66]. Here, fillets from fish supplemented with artemether showed lower pH values than the control group at the end of storage, suggesting delayed spoilage-related changes rather than a direct antioxidant effect alone. This response may be associated with the lower microbial counts observed in supplemented groups, especially psychrotrophic microorganisms, which are relevant spoilage bacteria under refrigerated conditions. Dietary supplementation with plant-derived bioactive compounds has been reported to influence post-harvest freshness indicators in fish fillets, including pH-related changes, oxidative stability, microbial development, and deterioration markers [61,62,63]. Therefore, the pH results should be interpreted together with microbial counts, TBARS values, colorimetric parameters, and sensory quality indicators, rather than as an isolated marker of preservation.

Color analysis in fish fillets is crucial for quality evaluation, as it directly affects visual appearance, sensory perception, and consumer acceptance [1,67]. In evaluating food color, the CIELAB color space (Commission Internationale de l’Eclairage) system is widely used due to its objective representation of color parameters, which is close to human visual perception [67,68]. In the colorimetric analysis carried out on the 15th day of storage, fillets from fish supplemented with artemether showed higher lightness values (L*) than the other treatments. This indicates that artemether-treated fillets were lighter, suggesting better preservation of visual quality during refrigerated storage. Lower lightness may be associated with progressive quality deterioration and darker appearance during storage. Because lipid oxidation and color deterioration are interconnected processes during fish storage, the higher lightness values observed in artemether-treated fillets may be interpreted together with the lower TBARS values and delayed deterioration indicators observed in this group [56,57].

Normally, a* values in tilapia fillets may decrease during storage as a consequence of post-harvest discoloration and oxidative changes in muscle pigments and lipids. On the 15th day of storage, fillets from the control group showed a significant shift toward greenish tones (negative a* values), while fillets from fish supplemented with *A. annua* powder displayed values closer to red tones (positive a* values), suggesting that color deterioration may have been less pronounced in this group. In the colorimetric evaluation of color intensity, ranging from yellow (positive b*) to blue (negative b*), fillets from fish supplemented with artemether presented lower negative b* values compared to the other treatments. After 30 days of refrigerated storage, all fillets showed a reduction in a* and an increase in b*, resulting in tones closer to green and yellow. The formation of free radicals during lipid oxidation and changes in muscle pigments may contribute to the reduction in a* values [69]. Surface color changes, including yellowing, may be associated with storage deterioration and lipid oxidation in refrigerated fish fillets [56,57,70]. Furthermore, the fillets showed a gradual reduction in the chroma saturation index (C*ab) values for all treatments up to 30 DPS, indicating a decrease in the vibrancy and purity of the fillets’ color over time. This change may be associated with refrigerated storage deterioration, including lipid oxidation as indicated by TBARS values and changes in colorimetric parameters. However, specific enzymatic reactions were not evaluated and therefore were not considered as a direct explanatory mechanism.

Delta E (ΔE) values in the CIELAB system represent the total color difference between two samples, providing an objective measure of the noticeable difference between them [68]. Analysis of the total color difference expressed as ΔE at 15 days of storage revealed significantly higher values (*p* < 0.05) for fillets from fish supplemented with artemether compared to the other groups. This indicates a noticeable color difference between groups and is consistent with the higher lightness values observed in artemether-treated fillets. Therefore, the ΔE results should be interpreted together with L*, a*, b*, chroma, and TBARS values as part of the overall visual and oxidative quality changes during refrigerated storage. Meanwhile, the hue angle (h°ab) values did not show significant differences, indicating that, despite variations in lightness and color intensity, the basic color tone of the fillets remained relatively stable among treatments.

In fresh fish, quality is often assessed through sensory-related characteristics, including appearance, brightness, texture, and odor. With the deterioration process, fish gradually loses its distinctive freshness attributes [1,71]. This phenomenon was observed here, where tilapia fillets stored under refrigeration showed a gradual decrease in brightness and texture, in addition to worsening appearance and odor. Fillets from fish supplemented with artemether showed better sensory quality scores, whereas fillets from the control group showed the lowest classifications. These sensory-related changes are consistent with the microbiological, physicochemical, TBARS, and colorimetric results, suggesting delayed quality deterioration in fillets from supplemented fish. Previous studies evaluating dietary supplementation strategies in fish have also reported effects on post-harvest fillet quality attributes during storage, including oxidative stability, freshness indicators, and sensory-related quality parameters [44,60,62,63]. However, because the sensory quality assessment was conducted with six trained panelists, these results should be interpreted as supportive evidence of fillet quality changes during refrigerated storage rather than as a full Quantitative Descriptive Analysis or consumer acceptance test. Future studies should include larger trained or consumer panels to confirm these sensory findings.

A limitation of the experimental design is the absence of a positive control, such as dietary vitamin E/α-tocopherol, BHT, or another standard antioxidant. Therefore, the effects observed for *A. annua*, artemisinin, and artemether should be interpreted in relation to the untreated control group, but not as evidence of superiority or equivalence to established antioxidant strategies. In addition, artemisinin and artemether are pharmacologically active compounds, and their residues were not quantified in edible tissues; therefore, bioavailability, muscle deposition, and residue depletion could not be determined. Direct antioxidant and antimicrobial activity assays of the tested materials were also not performed; consequently, the lower TBARS values and reduced counts of selected microbial groups should be interpreted as improvements in shelf-life-related indicators rather than as direct proof of intrinsic antioxidant or antimicrobial activity. Future studies should include standard antioxidant positive controls, residue quantification, dose–response evaluation, toxicological safety assessment, withdrawal period determination, and regulatory analysis before practical application in food-producing fish.

## 5. Conclusions

Dietary supplementation with *A. annua* powder (1%), artemisinin, or artemether (9.6 mg/kg feed) improved several shelf-life-related indicators of Nile tilapia fillets stored at 4 °C for up to 30 days, including microbial stability, lipid oxidation, and sensory quality, compared with the control. Among the tested treatments, artemether showed the most consistent effects, with lower microbial loads and oxidation indices for several evaluated parameters. Overall, these findings suggest that *A. annua*-derived compounds, especially artemether, may help delay post-harvest quality deterioration of refrigerated tilapia fillets. From a practical perspective, this dietary approach may represent a potential pre-harvest strategy to support refrigerated fish fillet quality and reduce losses associated with microbial spoilage and lipid oxidation. However, these results should be interpreted as preliminary and require further residue, tissue-deposition, and regulatory evaluation before any practical application in food-producing fish. Future studies should evaluate dose–response effects, tissue deposition, residue depletion, toxicological safety, withdrawal periods, and regulatory requirements, as well as include direct antioxidant assays and standard positive controls.

## Figures and Tables

**Table 1:** Correlation analysis between microorganism counts and pH values observed in tilapia fillets from different treatments.

Correlated Parameters	Experimental Sampling ^1^	Correlation Analysis	
		ρ ^2^	Prob > |ρ| ^2^
pH X Total coliforms	Artemether	0.28094	0.0875
	Artemisinin	0.64185	<0.0001
	*A. annua* powder	0.60842	<0.0001
	Control	0.71010	<0.0001
pH X Coagulase-positive staphylococci	Artemether	0.28935	0.0824
	Artemisinin	−0.02309	0.8953
	*A. annua* powder	0.43804	0.0047
	Control	0.57588	<0.0001
Mesophilic X Total microorganisms coliforms	Artemether	0.30067	0.0666
	Artemisinin	0.66463	<0.0001
	*A. annua* powder	0.76297	<0.0001
	Control	0.73386	<0.0001
Mesophilic X Coagulase-positive staphylococci	Artemether	0.31147	0.0606
	Artemisinin	−0.18989	0.2406
	*A. annua* powder	0.66693	<0.0001
	Control	0.63119	<0.0001
Psychrotrophic microorganisms X Coagulase-positive staphylococci	Artemether	0.25317	0.1363
	Artemisinin	0.06956	0.6868
	*A. annua* powder	0.50554	0.0010
	Control	0.58071	0.0002

^1^ Correlation analysis for fish treated with 9.6 mg artemether/kg feed (n = 40), fish treated with 9.6 mg artemisinin/kg feed (n = 40), fish treated with 1% *A. annua* powder (n = 40), and control fish (n = 40). ^2^ ρ = Spearman’s correlation coefficient; Prob. > |ρ| = significance probability of the ρ value.

**Table 2:** Correlation analysis between TBARS values and colorimetric parameters observed in tilapia fillets from different treatments.

Correlated Parameters	Experimental Sampling ^1^	Correlation Analysis	
		ρ ^2^	Prob > |ρ| ^2^
TBARS X Lightness (L*)	Artemether	0.65865	<0.0001
	Artemisinin	0.36853	0.0294
	*A. annua* powder	0.12807	0.4500
	Control	0.17394	0.2963
TBARS X Chroma	Artemether	−0.71052	<0.0001
	Artemisinin	−0.50431	0.0020
	*A. annua* powder	−0.50525	0.0014
	Control	−0.14318	0.3911
TBARS X Total color difference (ΔE)	Artemether	0.65576	<0.0001
	Artemisinin	0.35424	0.0368
	*A. annua* powder	0.12533	0.4598
	Control	0.16179	0.3318

^1^ Correlation analysis for fish treated with 9.6 mg artemether/kg feed (n = 40); fish treated with 9.6 mg artemisinin/kg feed (n = 40); fish treated with 1% *A. annua* powder (n = 40); and control fish (n = 40). ^2^ ρ = Spearman’s correlation coefficient; Prob. > |ρ| = significance probability of the ρ value.

## Data Availability

The original contributions presented in this study are included in the article. Further inquiries can be directed to the corresponding author.

## Abbreviations

APHA	American Public Health Association
BAM	Bacteriological Analytical Manual
BGLB	Brilliant Green Lactose Bile
BHI	Brain Heart Infusion
CAPES	Coordenação de Aperfeiçoamento de Pessoal de Nível Superior
CEUA	Ethics Committee on Animal Use
CFU/g	Colony-forming Units Per Gram
CIELAB	Commission Internationale de l’Eclairage Color System
CNPq	Conselho Nacional de Desenvolvimento Científico e Tecnológico
DPS	Days Post Slaughter
EC broth	*Escherichia coli* Broth
FAPESP	São Paulo Research Foundation
FDA	Food and Drug Administration
HPLC	High-performance Liquid Chromatography
LST	Lauryl Sulfate Tryptose
MPN	Most Probable Number
PCA	Plate Count Agar/Principal Component Analysis
PC1	Principal Component 1
PC2	Principal Component 2
PC3	Principal Component 3
PUFAs	Polyunsaturated Fatty Acids
ROS	Reactive Oxygen Species
SAS	Statistical Analysis Software
SPS Agar	Sulfite Polymyxin Sulfadiazine Agar
TBARS	Thiobarbituric Acid Reactive Substances
TBA	Thiobarbituric Acid
TCA	Trichloroacetic Acid
TLC	Thin-layer Chromatography
UNESP	São Paulo State University
UV	Ultraviolet
YSI	Yellow Springs Instruments
